# Expression of MTDH and IL-10 Is an Independent Predictor of Worse Prognosis in ER-Negative or PR-Negative Breast Cancer Patients

**DOI:** 10.3390/jcm9103153

**Published:** 2020-09-29

**Authors:** Pei-Yi Chu, Shin-Mae Wang, Po-Ming Chen, Feng-Yao Tang, En-Pei Isabel Chiang

**Affiliations:** 1 School of Medicine, College of Medicine, Fu Jen Catholic University, New Taipei 242, Taiwan; chu.peiyi@msa.hinet.net; 2 Department of Pathology, Show Chwan Memorial Hospital, Changhua 500, Taiwan; 3 Department of Health Food, Chung Chou University of Science and Technology, Changhua 510, Taiwan; 4 National Institute of Cancer Research, National Health Research Institute, Tainan 704, Taiwan; 5 Department of General Surgery, Show Chwan Memorial Hospital, Changhua 500, Taiwan; wangznmg@gmail.com; 6 Research Assistant Center, Show Chwan Memorial Hospital, Changhua 500, Taiwan; yaoming9@yahoo.com.tw; 7 Department of Food Science and Biotechnology, National Chung Hsing University, Taichung 40402, Taiwan; 8 Innovation and Development Center of Sustainable Agriculture (IDCSA), National Chung Hsing University, Taichung 402, Taiwan; 9 Biomedical Science Laboratory, Department of Nutrition, China Medical University, Taichung 40402, Taiwan; vincenttang@mail.cmu.edu.tw

**Keywords:** hypoxia, MTDH, IL-10, breast cancer, survival

## Abstract

(1) Background: Tumor hypoxia leads to metastasis and certain immune responses, and interferes with normal biological functions. It also affects glucose intake, down-regulates oxidative phosphorylation, and inhibits fatty-acid desaturation regulated by hypoxia-inducible factor 1α (HIF-1α). Although tumor hypoxia has been found to promote tumor metastasis, the roles of HIF-1α-regulated genes and their application are not completely integrated in clinical practice. (2) Methods: We examined the correlation between HIF-1α, metadherin (MTDH), and interleukin (IL)-10 mRNA expression, as well as their expression patterns in the prognosis of breast cancer using the Gene Expression Profiling Interactive Analysis (GEPIA) databases via a web interface; tissue microarrays (TMAs) were stained for MTDH and IL-10 protein expression using immunohistochemistry. (3) Results: HIF-1α, MTDH, and IL-10 mRNA expression are highly correlated and strongly associated with poor prognosis. MTDH and IL-10 protein expression of breast cancer patients usually harbored negative estrogen receptor (ER) or progesterone receptor (PR) status, and late-stage tumors have higher IL-10 expression. With regard to MTDH and IL-10 protein expression status for using univariate and multivariate analysis, the results showed that the protein expression of MTDH and IL-10 in ER-negative or PR-negative breast cancer patients have the worse prognosis. (4) Conclusions: we propose a new insight into hypoxia tumors in the metabolism and immune evidence for breast cancer therapy.

## 1. Introduction

Breast cancer is one of the most commonly diagnosed cancers. From 2006 to 2015, breast cancer incidence increased by approximately 1.8% per year among Asian women [1]. Breast cancer is a highly heterogeneous disease that is the second leading cause of cancer death in female patients [1]. The American Society of Clinical Oncology/College of American Pathologist (ASCO/CAP) recommends routine immunohistochemistry (IHC) on the expression of estrogen receptor (ER), progesterone receptor (PR), and human epidermal growth factor receptor 2 (HER2) for the determination of the pathological features [2,3,4,5].

Hypoxia is a hallmark of cancer. Malignant cancer cells cause an increase of anaerobic adenosine triphosphate (ATP) generation in response to a declined oxygen, rendering the aberrant vasculature development that leads to metastasis by increasing the transcription of hypoxia-inducible factor (HIF)-regulated genes [6,7]. HIF signaling acts as a central mediator, which enables inflammatory responses to recruit protumor immune cells, decreases cytotoxic antitumor CD8+ T cells, and alternatively activates M2 macrophages, as well as cellular metabolic reprogramming. HIF-1α-induced glycolysis metabolism is essential to the activation of inflammatory macrophage mediators [8]. Therefore, hypoxia-inducible factors have been suggested to be a central link between inflammation and cancer [7,8,9,10]. 

Experimental approaches, including a luciferase reporter assay, electrophoretic mobility shift assay (EMSA), and chromatin immuneprecitation (ChIP), have identified HIF-1α target genes, including immune response genes (C-X-C motif chemokine 12 (CXCL12), C-C motif chemokine ligand 2 (CCL2)), metastatic genes (vascular endothelial growth factor (VEGF), matrix metallopeptidase 2 (MMP2)), and metabolic gene (lysyl oxidase (LOX)) in various tumors [11]. Recently, metadherin (MTDH, also known as AEG-1 or Lyric), located on human chromosome 8q22, has been identified as an oncogene that can be regulated by HIF-1 signaling pathways in head and neck squamous cell carcinoma (HNSCC) [7]. HIF-1α can bind to the MTDH promoter and induce MTDH expression that enhances metastatic capabilities in HNSCC [7]. Hypoxia-mediated metastasis and glycolysis was interrupted by MTDH gene knockdown in HNSCC [7]. The expression of MTDH in breast cancer is related to the poor pathological features, poor prognosis, and chemo-resistance [12,13].

HIF-1α is a critical transcription factor for interleukin 10 (IL-10)-producing B cells in autoimmune diseases [14]. Macrophages usually accumulate in hypoxic sites, where they significantly impact disease progression, including cancer. Tumor-derived cytokines with immunosuppressive activities, such as IL-10 and IL-4, are able to convert tumor-associated macrophages (TAMs) to polarized type 2 (M2 macrophages) that promote cancer progression [15,16,17,18]. Anti-inflammatory cytokine IL-10 can generate alternatively-activated macrophages that support tumor growth [19]. In lung adenocarcinoma, IL-10 promotes tumor aggressiveness via the upregulation of CIP2A transcription [15]. IL-10 stimulates the expression of carboxypeptidase B2 and promotes lymphovascular invasion in inflammatory breast cancer cell-line SUM-149, but not in non-inflammatory breast cancer cell MDA-MB-231 [19]. These studies suggested potential roles of HIF-1α target genes in breast cancer and tumor hypoxia, and that HIF-1α-induced genes could be potential therapeutic targets for breast cancer. 

Currently, there are no protein expression disposition datasets established for breast cancer. In the present study, we explored the clinical significance of HIF-1α target gene MTDH, IL-10, CXCL12, CCL2, VEGF, MMP2, LOX, and C-X-C chemokine receptor type 4 (CXCR4) in breast cancer, using RNA-seq datasets from open sources, and further validated these findings in our own breast cancer cohort by immunohistochemistry. This is the first study that has evaluated the relationship between MTDH, IL-10, CXCL12, CCL2, VEGF, MMP2, LOX, CXCR4, and HIF-1α expression, and has also integrated such relationships into the overall survival of breast cancer using RNA-seq datasets. Using multiple approaches, we identified that MTDH and IL-10 protein expression is an independent predictor of worse prognosis in ER-negative and PR-negative breast cancer patients, which could predict overall survival.

## 2. Materials and Methods

### 2.1. Patients

Contralateral primary breast tumor and adjacent normal breast tissues of 265 breast cancer patients receiving surgical resection were acquired from Changhua Show Chwan Memorial Hospital from March 2011 to January 2017. Computed tomography (CT) was applied for the diagnosis in the 265 breast cancer patients prior to surgery. The diagnosis parameters and clinical outcomes were recruited until patient death or loss to follow-up. In the study, donor records were obtained from the Cancer Registry of Changhua Show Chwan Memorial Hospital. All personal identification information had been deleted and anonymized before we accessed the records, and personal privacy was under protection against using these data. The age of all patients was between 29 and 95 years old (mean ± SD: 54.88 ± 12.32 years). Clinical parameters and survival data were recorded from the cancer registry system of Changhua Show Chwan Memorial Hospital, which is anonymously linked to the Taiwan Cancer Registry (http://www.iacr.com.fr/index.php?option=com_comprofiler&task=userprofile&user=1440&Itemid=498). The variables included age, gender, tumor size, *N* (lymph nodes), *m* (metastasis), stage, ER, PR, HER2 status, date of operation, diagnosis, death, etc. Survival data was annotated to be the time from the date of primary surgery to the date of death. During this survey, 29 patients died and 52 patients exhibited tumor metastasis. The metastasis sites included the skin, abdomen, pleura, bone, lung, liver, chest wall, breast, and lymph node. The median overall survival time of all breast cancer patients was 1440 days. This project was approved by the Ethics Committee of the Institutional Review Board of Show Chwan Memorial Hospital (IRB No. 1060407).

### 2.2. Immunohistochemistry and Scoring 

For each patient, representative tissue cores of the breast tumor section, as well the adjacent normal section, were carefully collected and made into tissue microarrays. Immunohistochemistry (IHC) staining was used to evaluate MTDH and IL-10 protein expression. The MTDH antibody (Abcam, ab104836) and IL-10 antibody (Abcam, ab34843) were purchased from Abcam (Cambridge, MA, United States). IHC evaluation and protocol used to obtain score have been descripted previously [20]. The average signals of the scores were evaluated independently by two pathologists that were blinded when judging the slides. Immunostaining scores were defined as the cell staining intensity (0 = none; 1 = weak; 2 = moderate; and 3 = strong) multiplied by the percentage of labeled cells (0% to 100%), leading to scores from 0 to 300. The mean of score of the signals were evaluated independently by two pathologists. Immunostaining scores were defined as the cell staining intensity levels included (0 = none, 1 = weak, 2 = moderate, and 3 = strong) multiplied by the proportion of stained cells (0% to 100%), leading to scores ranging from 0 to 300. The IHC staining median score was used as the cut-off point for the dichotomization of MTDH and IL-10. A score more than the median was recognized as “high” immunostaining, whereas a score less than or equal to the median was recognized as “low”. 

### 2.3. Statistical Analysis

The association between MTDH and IL-10 protein expression and the clinical and pathological parameters was calculated using a Chi-squared test, and Pearson’s correlation was used for the association between MTDH and IL-10 protein expression. Survival curves were plotted using the Kaplan–Meier model and compared using a log–rank test. Additionally, Cox’s proportional hazards regression model was used to analyze the association between age, stage, MTDH, IL-10, and survival data, and *p* < 0.05 was considered to indicate a statistically significant difference. SPSS 18.0 software (SPSS, Inc., Chicago, IL, USA) was used for all statistical analyses.

### 2.4. Web Server Survival Analysis 

The correlation of expression of MTDH and IL-10 mRNA was calculated using Pearson’s correlation. The survival analysis of MTDH, IL-10, and HIF-1α mRNA expression in this study was performed using the web server for the Kaplan–Meier plots from RNA-seq datasets by auto selecting the median values between the lower and upper quartiles into high and low expression. Please have a look at http://gepia2.cancer-pku.cn/#index.

## 3. Results

### 3.1. Exploring the Correlation and Clinical Significance of HIF-1α, MTDH, IL-10, CCL12, CCL2, VEGF, MMP2, LOX, and CXCR4 Expression in Breast Cancer

**Figure 1:**
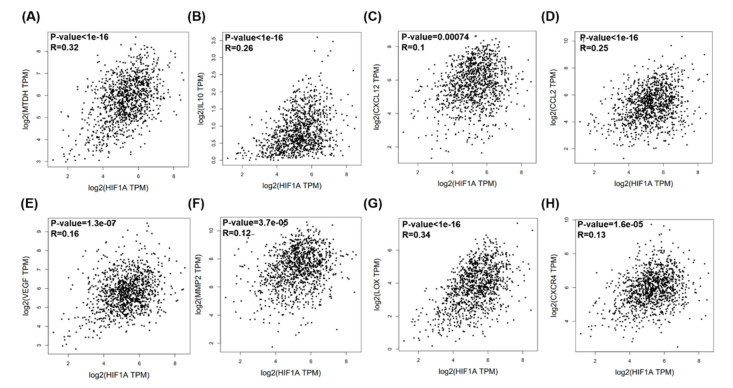
Metadherin (MTDH), interleukin (IL)-10, C-C motif chemokine ligand 12 (CCL12), C-C motif chemokine ligand 2 (CCL2), vascular endothelial growth factor (VEGF), matrix metallopeptidase 2 (MMP2), lysyl oxidase (LOX), and C-X-C chemokine receptor type 4 (CXCR4) mRNA expressions and their correlations with hypoxia-inducible factor (HIF)-1α in breast cancer patients using the The Cancer Genome Atlas (TCGA) web server program. Pearson’s correlations between HIF1-α expression and (**A**) MTDH, (**B**) IL-10, (**C**) CXCL12, (**D**) CCL2, (**E**) VEGF, (**F**) MMP2, (**G**) LOX, and (**H**) CXCR4 expressions.

**Figure 2:**
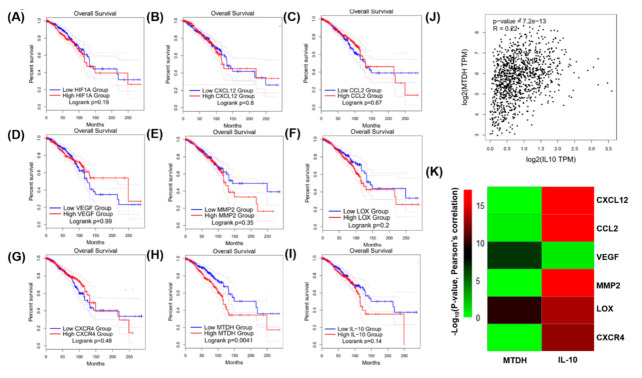
Clinical prognosis of breast cancer patients with the expressions of HIF-1α and its target genes (MTDH, IL-10, CCL12, CCL2, VEGF, MMP2, LOX, and CXCR4) using the TCGA web server program. The Kaplan–Meier plot shows that lower expressions of HIF1-α, MTDH (**H**), and IL-10 (**I**) were modestly associated with better overall survival of breast cancer patients. CXCL12, CCL2, VEGF, MMP2, and CXCR4 expression was not associated with overall survival of breast cancer patients. (**A**–**G**). (**J**) Pearson’s correlation elucidated a strong association between MTDH and IL-10 expression. (**K**) Heatmap of Pearson’s correlations, shown by calculating (−Log10(*p*-value))) for MTDH and IL-10 use with CXCL12, CCL2, VEGF, MMP2, LOX, and CXCR4.

Breast cancer clinical data and RNA-seq data were collected from The Cancer Genome Atlas (TCGA) datasets and analyzed by Gene Expression Profiling Interactive Analysis (GEPIA) [21]. Correlation and overall survival analyses of MTDH, IL-10, CXCL12, CCL2, VEGF, MMP2, LOX, CXCR4, and HIF-1α mRNA expression were performed using GEPIA plotters. The results revealed that MTDH, IL-10, CXCL12, CCL2, VEGF, MMP2, LOX, and CXCR4 mRNA expression was significantly correlated with HIF1-α mRNA expression (Figure 1). Among the HIF-1α target genes, MTDH and IL-10 mRNA expression was correlated with HIF-1α, MTDH, and IL-10, with smaller *p*-values than others in clinical prognosis (*p* = 0.0041 and *p* = 0.14, respectively; Figure 2H,I). Pearson’s correlation coefficient revealed that MTDH expression was positively associated with IL-10 (*p* = 7.2 × 10^13^, correlation coefficient (*R*) = 0.22, Figure 2J). The heatmap revealed that VEGF and LOX mRNA expression were correlated with MTDH mRNA expression, and CXCL12, CCL2, MMP2, LOX, and CXCR4 mRNA expression were correlated with IL-10 mRNA expression (Figure 2K).

MTDH and IL-10 were ultimately selected for immunohistochemistry analyses based on their associations with poor survival (*p* = 0.0041 in Figure 1E, and *p* = 0.14 in Figure 1F, respectively). The survival curves indicated that high expression of MTDH was associated with poor overall survival (Figure 1E). A similar trend was observed in patients with high HIF-1α and IL-10 expression who appeared to have poor overall survival (Figure 1C,F). Although CXCL12, CCL2, VEGF, MMP2, LOX, and CXCR4 were highly associated with HIF-1α expression (Figure 1), their prognostic significance in breast cancer is less (Figure 2) so they were not chosen for further immunohistochemistry analyses.

### 3.2. MTDH, and IL-10 Protein Expression Are Positively Correlated with Hormone Receptor Protein Expression

**Figure 3:**
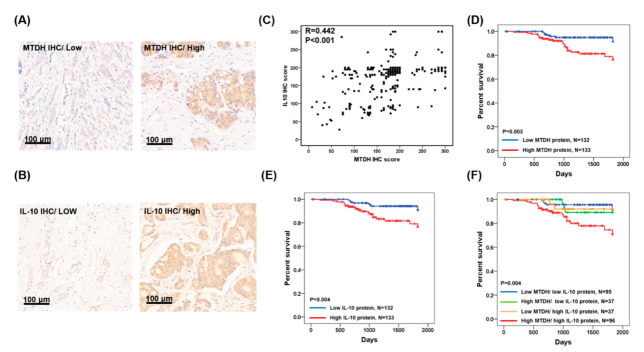
MTDH and IL-10 protein expression in tumor tissues of breast cancer patients, and Kaplan–Meier analysis of MTDH and IL-10 protein expression for breast cancer patients. (**A**) Representative low and high MTDH immunostaining results in breast cancer tissues. (**B**) Representative low and high IL-10 immunostaining results in breast cancer tissue. (**C**) Pearson’s correlation used to elucidate MTDH expression in relation to IL-10 protein expression. (**D**) Overall survival estimates for MTDH. (**E**) Overall survival estimates for IL-10. (**F**) Overall survival estimates for MTDH/IL-10.

To verify whether MTDH and IL-10 protein expressions were linked with clinicopathological parameters of breast cancer, we enrolled 265 breast cancer patients and performed immunohistochemistry of MTDH and IL-10. MTDH and IL-10 expression were observed in the cytoplasm of the tumor sections. Representative results are shown in Figure 3A,B. Meanwhile, the protein expressions of MTDH and IL-10 were positively correlated in our breast cancer cohort (*R* = 0.442, *p* < 0.001, *n* = 265; Figure 3C). Of the 265 tumors, the expression levels of MTDH in the ER-negative or PR-negative tumor tissue were significantly higher than that in the ER-positive or PR-positive tumors (*p* = 0.007 and *p* = 0.014, respectively; Table 1), but no significant correlation was found in those over age 65 (*p* = 0.656; Table 1), with late-stage tumors (*p* = 0.092; Table 1), or with HER2-positive tumors (*p* = 0.379; Table 1). The expression level of IL-10 in the ER-negative or PR-negative tumor tissue was significantly higher in ER-positive or PR-positive tumors (*p* = 0.014 and *p* = 0.007, respectively; Table 1), but no significant correlation was found in those over aged 65 (*p* = 0.446; Table 1) or with HER2-positive tumors (*p* = 0.929; Table 1). Interestingly, the expression levels of IL-10 in the late-stage (stage III and stage IV) tumor tissue were significantly higher in early-stage (stage I and stage II) tumors (*p* = 0.046; Table 1). 

### 3.3. Expression of MTDH and IL-10 Protein Are Associated with Poor Prognosis of Breast Cancer

The MTDH, IL-10, age, stage, ER status, PR status, and HER2 status were selected as prognostic markers by univariate analysis. The MTDH, IL-10, age, stage, ER status, and PR status significantly correlated with the five-year survival rate (*p* = 0.003, *p* = 0.004, *p* = 0.024, *p* < 0.0001, *p* = 0.002, and *p* = 0.018; respectively; Table 2). Kaplan–Meier analysis showed that patients with high MTDH expression had shorter five-year overall survival periods when compared to that of patients with low MTDH expression (*p* = 0.003; Figure 3D). Similarly, patients with high IL-10 expression had shorter five-year overall survival periods (*p* = 0.004; Figure 3E). In addition, patients with simultaneously high MTDH and IL-10 had the worst five-year survival rate (*p* = 0.004; Table 2, Figure 3F).

Cox regression analysis further indicated a prognostic significance of MTDH and IL-10 expression, age, and stage on five-year overall survival period in breast cancer (Table 3). The hazard ratios of the high MTDH expression in combination with high IL-10 was 8.0 for the five-year survival rate when the low expression of MTDH and IL-10 was used as a reference (Table 3). Additionally, the hazard ratios for late-stage was 34.25 for the five-year survival rate, when early-stage was used as a reference (Table 3). The hazard ratio for the five-year overall survival period for patients over age 65 was not statistically significant from that of those below 65 (Table 3).

**Figure 4:**
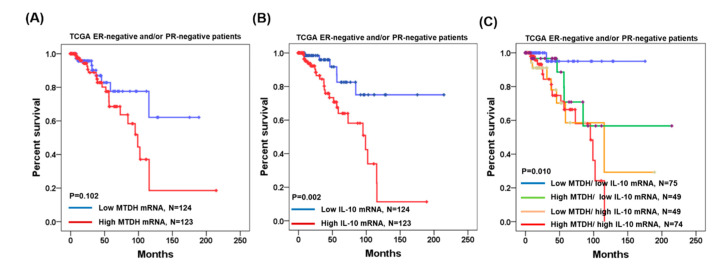
Kaplan–Meier analysis of MTDH and IL-10 mRNA expressions in 247 ER-negative and/or PR-negative breast cancer patients using TCGA. (**A**) Overall survival estimates for MTDH expression. (**B**) Overall survival estimates for IL-10 expression. (**C**) Overall survival estimates for MTDH/IL-10 expression.

We have collected MTDH and IL-10 mRNA expression data and clinical parameters from OncoLnc (http://www.oncolnc.org/search_results/?q=HIF1A-HS1 and https://www.oncomine.org/resource/login.html). In the 247 ER-negative and/or PR-negative breast cancer patients, Kaplan–Meier analysis showed that patients with high MTDH mRNA expression had shorter overall survival periods when compared to those of patients with low MTDH expression (*p* = 0.102; Figure 2A). Similarly, patients with high IL-10 mRNA expression had significantly shorter overall survival periods (*p* = 0.002; Figure 4B). In addition, patients with simultaneously high MTDH and IL-10 had the worse survival rate (*p* = 0.010; Figure 4C).

## 4. Discussion

A large prospective cohort of breast cancer survivors indicated that the main comorbidities included hypertension, chronic gastritis, diabetes mellitus, chronic bronchitis/asthma, coronary heart disease, and history of stroke. Diabetes was significantly associated with increased risk of total and non-breast cancer mortality, and history of stroke was associated was associated with increased risk of non-breast cancer mortality [22]. In the study, the above comorbidity information was not available, but we were able to collect important information anonymously linked to the Taiwan Cancer Registry including age, gender, tumor size, lymph node invasion, metastasis, stage, ER, PR, HER2 status, date of operation, diagnosis, and death.

The molecular diagnosis markers for breast cancer have not been fully elucidated. In our cohort involving 265 breast cancer patients, MTDH and IL-10 expression significantly correlated with clinically characteristic ER and PR status (Table 1). Elevations in IL-10 is also associated with later stages. These findings are comparable with other breast cancer studies showing that MTDH overexpression is associated with an aggressive phenotype and a poor prognosis in breast cancer [23]. On the other hand, IL-10 expression was reported to be associated with good prognosis in early-stage invasive breast cancer patients (non-triple-negative breast cancer (non-TNBC)) [24]. In contrast, multivariate analysis demonstrated that a higher IL-10 level is associated with worse disease conditions, including late-stage, ER-negative, and PR-negative breast cancer in our study. A systematic review and meta-analysis revealed that the rs1800871 and rs1800872 polymorphisms of the IL-10 gene are associated with overall breast cancer risk in the general population [25]. We speculate that IL-10 expression in breast cancer may serve as a preferential metastatic condition that permits cells to evade host anticancer immunity, which is dependent on the ER and PR status. If so, in contrast to the ER-positive and PR-positive breast cancer that can be treated with a mainstay hormone therapy, immune therapy should be developed for ER-negative and PR-negative breast cancer with tamoxifen resistance [26].

MTDH is an oncoprotein in numerous human tumorigeneses, including lung, colon, head and neck, liver, glioma, and breast cancers [27]. In HNSCC, hypoxia promoted glucose uptake and lactate production, and induced cell metastasis, glycolysis, MTDH expression in HNSCC cell Tu686 [7]. Hypoxia promotes cell invasion, tumor metastasis, and epithelial–mesenchymal transition by mediating the HIF-1α–MTDH loop in HNSCC cells [7]. Our present study indicates that MTDH is related to aggressive phenotypes and a poor prognosis; hence, it is a potential target for anticancer drugs in breast cancer.

The exact mechanism and functional significance of MTDH-mediated glycolysis and metastasis in breast cancer remains to be investigated further. Although MTDH is obviously expressed in the cytoplasm in breast tumors, the existence of a nuclear MTDH expression in prostate cancer and NIH3T3 cells has been reported previously [24,28,29]. Changes in its subcellular distribution can predict Gleason grade and survival in prostate cancer patients. The nuclear localization of MTDH protein has three putative, lysine-rich NLS sequences in the prostate tissue [28] that might result from RNA splicing in different tumor types. Two lysine-rich regions (NLS-1 and NLS-3) can target MTDH to subcellular compartments, whereas NLS-2 is modified by ubiquitin in the cytoplasm. Molecular interactions between MTDH and many effector molecules of signal transduction pathways, including Wnt/β-catenin, PI3K/AKT, NF-ΚB, CLDN4/tetrasponin 8, IGFBP-7, and MYC-mediated processes can lead to cell growth, invasion, metastasis, and senescence [30]. Despite that, IL-10 acts a multifunctional, immune-regulatory cytokine with both immunosuppressive and anti-angiogenic functions in immune cells (macrophages, T lymphocytes, and natural killer cells). IL-10 promotes breast cancer cell proliferation and metastasis via immunosuppression [31]. More studies on the mechanism and functional significance of MTDH- and IL-10 mediated breast cancer progression are warranted.

In summary, we demonstrated the clinical significance of HIF-1α-regulated genes, MTDH, and IL-10 using open source data and our cohort study. Not all HIF-1α-regulated genes are included in our study, yet we identified two potential targets in the metabolic and immune systems that could be critical in breast cancer progression. Particularly, the combination of high MTDH and high IL-10 expression is a dependent prognosis factor in ER-negative and/or PR-negative breast cancer. Our study suggests that the inhibition of MTDH and IL-10 may have potential with regard to developing new therapeutic strategies for breast cancer.

## Figures and Tables

**Table 1:** Relationship of Clinical Parameters with MTDH and IL-10 protein expression in 265 breast cancer.

		MTDH				IL-10	
Characteristics	No.	Low (*n* = 132)	High (*n* = 133)	*p*-Value	Low (*n* = 132)	High (*n* = 133)	*p*-Value
Age							
<65	216	109 (51)	107 (49)	0.656	110 (51)	106 (49)	0.446
≥65	49	23 (47)	26 (53)		22 (45)	27 (55)	
Stage							
I, II	214	112 (52)	102 (48)	0.092	113 (53)	101 (47)	0.046
III, IV	51	20 (39)	31 (61)		19 (39)	32 (61)	
estrogen receptor (ER)							
Negative	76	28 (37)	48 (63)	0.007	25 (33)	51 (67)	<0.001
Positive	189	104 (55)	85 (45)		107 (57)	82 (43)	
progesterone receptor (PR)							
Negative	106	43 (41)	63 (59)	0.014	42 (40)	64 (60)	0.007
Positive	159	89 (56)	70 (44)		90 (57)	69 (43)	
human epidermal growth factor receptor 2 (HER2)							
Negative	180	93 (52)	87 (48)	0.379	90 (50)	90 (50)	0.929
Positive	85	39 (46)	46 (54)		42 (49)	43 (51)	

Chi-squared test for *p*-value.

**Table 2:** Univariate analysis of influence of clinical characteristics on overall survival in 265 breast carcinoma patients.

		Overall Survival (OS)		
Characteristics	No.	Median Survival (Days)	5-Year Survival (%)	Log-Rank
Age				
<65	216	1534	195 (91)	0.024
≥65	49	1353	40 (82)	
Stage				
I, II	214	1541	204 (95)	<0.001
III, IV	51	981	32 (63)	
ER				
Negative	76	1231	61 (80)	0.002
Positive	189	1569	175 (93)	
PR				
Negative	106	1231	89 (84)	0.018
Positive	159	1624	147 (93)	
HER2				
Negative	180	1440	165 (92)	0.076
Positive	85	1492	71 (88)	
MTDH				
Low	132	1805	124 (94)	0.003
High	133	1100	112 (84)	
IL-10				
Low	132	1734	123 (93)	0.004
High	133	1073	113 (86)	
MTDH/IL-10				
Low/low	95	1825	89 (94)	0.004
Low/high	37	1825	34 (92)	
High/low	37	1825	35 (95)	
High/high	96	1057	78 (81)	

Log rank test for *p*-value.

**Table 3:** Cox regression analysis for the influence of age, stage, and MTDH/IL-10 on overall survival in 107 ER-negative and/or PR-negative breast carcinoma patients.

		Overall Survival (OS)		
Characteristics	HR	Unfavorable/Favorable	*p*-Value	95% CI
Age	3.00	≥65/<65	0.154	0.66–13.34
Stage	34.25	III, IV/I, II	<0.001	8.25–142.29
MTDH/IL-10	8.00	High, high/low, and low	0.023	1.34–48.06

Cox regression model for age, stage, and MTDH/IL-10.
